# Differential Gene Expression in Synovium Between Male and Female Knee Osteoarthritis

**DOI:** 10.3390/medicina62071338

**Published:** 2026-07-11

**Authors:** Akira Norisugi, Kentaro Uchida, Kensuke Fukushima, Manabu Mukai, Yoshihisa Ohashi, Yui Uekusa, Ayumi Tsukada, Dai Iwase, Jun Aikawa, Yukie Metoki, Gen Inoue, Masashi Takaso

**Affiliations:** 1 Department of Orthopaedic Surgery, Kitasato University School of Medicine, 1-15-1 Minami-ku, Kitasato, Sagamihara 252-0374, Kanagawa, Japan; igusiron@gmail.com (A.N.); kenfu@r4.dion.ne.jp (K.F.); m.manabu0829@hotmail.co.jp (M.M.); 44134413oo@gmail.com (Y.O.); uekusa18y@gmail.com (Y.U.); amidesu-tarere9010@yahoo.co.jp (A.T.); daiiwase19760601@yahoo.co.jp (D.I.); jun43814@gmail.com (J.A.); yukiemetoki0826@gmail.com (Y.M.); ginoue@kitasato-u.ac.jp (G.I.); mtakaso@kitasato-u.ac.jp (M.T.); 2 Research Institute, Shonan University of Medical Sciences, Nishikubo 500, Chigasaki 253-0083, Kanagawa, Japan

**Keywords:** sex difference, osteoarthritis, synovium

## Abstract

*Background and Objectives*: Sex differences are well recognized in the epidemiology and clinical manifestations of knee osteoarthritis (OA), with women exhibiting a higher prevalence and greater disease severity than men. Although synovial inflammation is increasingly recognized as a key contributor to OA pathology, the molecular mechanisms underlying sex-related differences in OA synovium remain incompletely understood. *Materials and Methods*: Synovial tissues were obtained from patients with knee OA undergoing total knee arthroplasty. RNA sequencing (RNA-seq) was initially performed using synovial samples from five female and five male patients to identify differentially expressed genes (DEGs) associated with sex. Candidate genes identified by RNA-seq were subsequently validated by quantitative PCR (qPCR) using an independent cohort consisting of 78 female and 27 male patients. Multivariable analyses adjusted for age, body mass index (BMI), and Kellgren–Lawrence (KL) grade were performed to evaluate the independent association between gene expression and sex. *Results*: RNA-seq analysis identified 12 female-upregulated genes and 13 male-upregulated genes. Several Y chromosome-related genes showed marked male-specific expression and were excluded from downstream validation analyses. qPCR validation demonstrated significantly higher expression of *CAPN6*, *COL6A6*, *EGFL6*, *LAMP3*, and *MMD* in female synovial tissues. After adjustment for age, BMI, and KL grade, *EGFL6* (β = 0.829, *p* = 0.004), *LAMP3* (β = 0.596, *p* = 0.029), and *MMD* (β = 0.698, *p* = 0.014) remained significantly associated with female sex. In contrast, *DAW1* became significantly associated with male sex after multivariable adjustment (β = 0.753, *p* = 0.009). *Conclusions*: Distinct sex-related synovial gene expression profiles were identified in knee OA. In particular, *EGFL6*, *LAMP3*, and *MMD* were independently associated with female sex, suggesting potential sex-related pathology in OA synovium. These findings provide new insight into the molecular basis of sex differences in OA and may contribute to the development of sex-specific therapeutic strategies.

## 1. Introduction

Knee osteoarthritis (OA) is one of the most common musculoskeletal disorders and a leading cause of disability in older adults worldwide [1]. OA is characterized not only by progressive cartilage degeneration but also by synovial inflammation, subchondral bone remodeling, and alterations in periarticular tissues [2,3]. Increasing evidence suggests that the synovium plays a pivotal role in OA pathogenesis through the production of inflammatory mediators, angiogenic factors, and extracellular matrix-remodeling enzymes [4,5].

In addition to synovitis, OA synovium can exhibit immune cell infiltration, increased vascularity, sublining fibrosis, and activation of synovial fibroblasts [4,5,6]. Synovial fibroblasts contribute to inflammatory, fibrotic, and extracellular matrix-remodeling responses through interactions with immune and other stromal cells [6,7]. These processes may contribute to OA progression and pain and provide a biological context in which sex-related molecular differences may arise.

Sex differences are well recognized in the epidemiology and clinical manifestations of OA. Women exhibit a higher prevalence and greater severity of knee OA, particularly after menopause [8,9]. Sex-related differences in OA are likely multifactorial and may involve interactions among hormonal status, immune responses, angiogenesis, and stromal remodeling. Changes in sex hormone signaling during the menopausal transition may influence inflammatory and tissue-remodeling processes within the joint; however, their effects are likely tissue- and context-dependent [10]. Recent studies, including analyses from our group, have identified sex-related molecular alterations in OA synovium, including increased expression of calcitonin gene-related peptide (CGRP), a neuropeptide associated with pain signaling, and IL24, an inflammatory cytokine, in female OA patients [11,12]. These findings suggest that sex-related molecular alterations may exist in OA synovium. However, sex-related molecular characteristics of OA synovium have not been fully characterized.

In the present study, we investigated sex-related differences in synovial gene expression in patients with knee OA using RNA sequencing and quantitative PCR analyses to characterize molecular features associated with sex-related differences in OA.

## 2. Materials and Methods

### 2.1. Study Participants

This study was conducted in accordance with the principles of the Declaration of Helsinki and was approved by the Institutional Review Board of Kitasato University using an opt-out consent approach (B19-259). Patients with knee OA diagnosed through clinical and radiographic assessment were enrolled in this study. Individuals with rheumatoid arthritis, autoimmune disorders, inflammatory joint diseases, systemic diseases affecting the joints, or previous joint replacement surgery were excluded. Synovial tissue samples were obtained from patients with radiographically confirmed knee OA who underwent total knee arthroplasty at our institution. During the surgical procedure, synovial tissues were harvested from the affected knee joint. A total of 115 synovial specimens were immediately snap-frozen in liquid nitrogen and subsequently stored at −80 °C until RNA extraction.

### 2.2. RNA-Seq

Total RNA was extracted from synovial tissue using a phenol/chloroform extraction method, ensuring high RNA yield and purity. RNA quantity was determined using a spectrophotometer (Denovix, Wilmington, DE, USA) and quality was assessed on an Agilent 2100 BioAnalyzer (Agilent Technologies, Santa Clara, CA, USA) with an RNA 6000 Nano Chip. RNA-Seq was conducted on extracted RNA. RNA sequencing was performed using an MGI DNBSEQ-G400 sequencer (BGI, Shenzhen, China). Differentially expressed genes (DEGs) were identified using a threshold of |log2 fold change| > 1.0 and q-value < 0.05.

### 2.3. qPCR

Quantitative PCR (qPCR) was performed in a 25 μL reaction volume containing 2 μL of cDNA, 12.5 μL of TB Green Premix Ex Taq II (Tli RNaseH Plus; Takara Bio, Shiga, Japan), 2 μL of forward–reverse primer mixture, and 8.5 μL of nuclease-free water. Primers were designed using Primer-BLAST (NCBI) and synthesized by Hokkaido System Science (Hokkaido, Japan). Reactions were run in triplicate using the CFX96 Real-Time PCR Detection System (Bio-Rad, Hercules, CA, USA) under the following cycling conditions: initial denaturation at 95 °C for 3 min, followed by 40 cycles of denaturation at 95 °C for 10 s and annealing/extension at 60 °C for 30 s. Primer sequences are listed in Table 1. Gene expression levels were normalized to GAPDH as an internal control, and relative expression levels were calculated using the 2^−ΔΔCt^ method, with the mean expression level in male samples set to 1.0 as the reference. Amplification specificity was confirmed by melt curve analysis.

### 2.4. Statistical Analysis

Data were analyzed using SPSS software (Version 28.0; IBM Corp., Armonk, NY, USA). Normality of continuous variables was assessed using the Shapiro–Wilk test. To ensure consistent presentation across cohorts, continuous variables are presented as median (range). Age was normally distributed in both cohorts, and BMI was normally distributed in the RNA-seq cohort; these variables were compared between male and female groups using Student’s *t*-test. BMI in the qPCR cohort and gene expression data were non-normally distributed and were compared using the Mann–Whitney U test. Categorical variables, including Kellgren–Lawrence grade, are presented as number (percentage) and were compared using Fisher’s exact test. To assess the independent association between gene expression and sex after adjustment for clinical covariates, multivariable linear regression analysis was performed with sex as the independent variable and age, BMI, and KL grade included as covariates. A *p*-value < 0.05 was considered statistically significant.

## 3. Results

### 3.1. RNA-Seq Analysis of Sex-Related Differences in Synovial Gene Expression

Table 2 summarizes the demographic and clinical characteristics of the study participants. No significant differences in age, BMI, or KL grade distribution were observed between female and male participants in either cohort.

**Figure 1:**
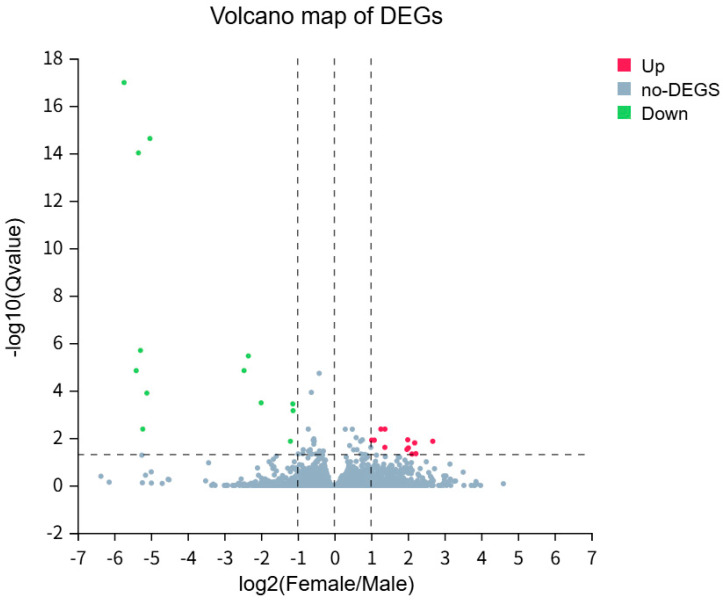
Volcano plot showing differentially expressed genes (DEGs) between female and male synovial tissues identified by RNA-seq analysis. The x-axis represents log2 fold change (Female/Male), and the y-axis represents −log10(q-value). Red dots indicate genes significantly upregulated in females, whereas green dots indicate genes significantly downregulated in females (male-upregulated genes). Gray dots represent non-significant genes. Dashed vertical lines indicate fold-change thresholds, and the horizontal dashed line indicates the significance threshold (q-value < 0.05).

To explore sex-related differences in synovial gene expression, RNA-seq was performed using synovial tissue samples obtained from five female and five male patients. Volcano plot analysis demonstrated distinct sex-related gene expression profiles between female and male samples (Figure 1). Using a threshold of |log2 fold change| > 1.0 and q-value < 0.05, 12 female-upregulated genes and 13 male-upregulated genes were identified (Table 3).

Among the female-upregulated genes, *APCDD1L*, *AZGP1*, *C3orf80*, *CAPN6*, *CDK1*, *COL6A6*, *EGFL6*, *FMO1*, *LAMP3*, *LEF1*, *LRRN1*, and *MMD* were selected for subsequent validation based on expression levels, statistical significance. In male samples, several Y chromosome-related genes, including *KDM5D*, *DDX3Y*, *ZFY*, *USP9Y*, *RPS4Y1*, *EIF1AY*, and *NLGN4Y*, showed markedly increased expression. In addition, *ENSG00000266302*, an uncharacterized Ensembl transcript showing male-specific expression, was also highly expressed in males. Because these genes primarily reflected chromosomal sex differences, they were excluded from downstream validation analyses. Consequently, *DACT2*, *DAW1*, *DUOX2*, *P2RY2*, and *USP2* were selected for validation.

### 3.2. Gene Expression Analysis by qPCR

**Figure 2:**
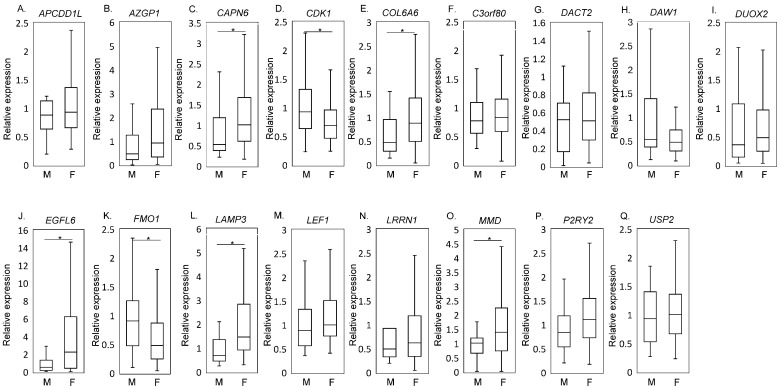
Sex-related differences in synovial gene expression assessed by qPCR. Box-and-whisker plots showing the relative expression levels of *APCDD1L* (**A**), *AZGP1* (**B**), *CAPN6* (**C**), *CDK1* (**D**), *COL6A6* (**E**), *C3orf80* (**F**), *DACT2* (**G**), *DAW1* (**H**), *DUOX2* (**I**), *EGFL6* (**J**), *FMO1* (**K**), *LAMP3* (**L**), *LEF1* (**M**), *LRRN1* (**N**), *MMD* (**O**), *P2RY2* (**P**), and *USP2* (**Q**) in synovial tissue from male (M) and female (F) patients with knee osteoarthritis. Boxes represent the interquartile range, horizontal lines indicate the median, and whiskers indicate the minimum and maximum values. Asterisks indicate statistically significant differences between sexes in unadjusted comparisons (* *p* < 0.05).

The demographic and clinical characteristics of the qPCR validation cohort are presented in Table 2. The cohort included patients with varying KL grades representing different stages of OA severity, and no significant sex-related differences in age or BMI were observed between groups. To validate the RNA-seq findings, qPCR analysis was performed using an independent cohort of synovial tissue samples (Figure 2). In the unadjusted analysis, *CAPN6*, *COL6A6*, *EGFL6*, *LAMP3*, and *MMD* showed significantly higher expression in females, consistent with the RNA-seq results. In contrast, *CDK1* and *FMO1* showed significantly higher expression in males, which was inconsistent with the RNA-seq findings. Among the female-upregulated genes, *APCDD1L*, *AZGP1*, *C3orf80*, *LEF1*, and *LRRN1* did not show significant sex-related differences in the unadjusted analysis. Similarly, among the male-upregulated genes, no significant sex-related differences were observed for *DACT2*, *DAW1*, *DUOX2*, *P2RY2*, or *USP2*.

To further evaluate the independent association between gene expression and sex, multivariable linear regression analysis adjusted for BMI, age, and KL grade was performed (Table 4). Among the female-upregulated genes, *EGFL6* (β = 0.829, *p* = 0.004), *LAMP3* (β = 0.596, *p* = 0.029) and *MMD* (β = 0.698, *p* = 0.014) remained significantly associated with female sex, whereas *CAPN6* (β = 0.401, *p* = 0.088) and *COL6A6* (β = 0.138, *p* = 0.387) were no longer significant after adjustment. Among the male-upregulated genes identified by RNA-Seq, *DAW1* became significantly associated with male sex after adjustment for confounding factors (β = 0.753, *p* = 0.009), although no significant difference was observed in the unadjusted analysis. In contrast, *CDK1* was significantly associated with male sex despite showing female-upregulated gene expression in RNA-seq analysis. No significant associations were observed for age or KL grade in most genes, whereas BMI showed significant associations with *AZGP1* (β = 0.639, *p* = 0.021) and *EGFL6* (β = 0.506, *p* = 0.049) expression.

## 4. Discussion

In the present study, we identified distinct sex-related synovial gene expression profiles in patients with knee OA using RNA-seq analysis. Female synovial tissues demonstrated increased expression of several genes associated with angiogenesis, extracellular matrix remodeling, and inflammatory regulation, whereas male samples exhibited higher expression of a limited number of genes after exclusion of Y chromosome-related transcripts. Subsequent qPCR validation and multivariable regression analysis further demonstrated that some of these differences remained significant after adjustment for age, BMI, and KL grade, suggesting that biological sex independently influences synovial molecular signatures in OA.

Among the female-upregulated genes identified in the present study, *EGFL6*, *LAMP3*, and *MMD* remained significantly associated with female sex after multivariable adjustment. EGFL6 is an epidermal growth factor-like protein known to promote endothelial cell migration and angiogenesis through ERK signaling activation [13]. In addition, experimental OA models have demonstrated that EGFL6 is consistently upregulated during early OA development. In a rat meniscal tear model, EGFL6 expression was increased at multiple time points after OA induction and was categorized among genes associated with extracellular matrix remodeling and angiogenesis [14]. Pathway analyses further identified angiogenesis and vasculature development as key biological processes shared between experimental OA models and human OA cartilage, suggesting that angiogenic signaling is an important component of OA pathogenesis. Collectively, these findings suggest that increased *EGFL6* expression in female synovium may contribute to sex-related differences in OA pathology.

LAMP3 was also identified as a female-upregulated gene in the present study. Previous transcriptomic analyses comparing rheumatoid arthritis (RA) and OA synovial tissues identified *LAMP3* as one of the differentially expressed genes enriched in inflammatory and immune-related pathways [15]. In addition, increased *LAMP3* expression has been reported in RA synovium and fibroblast-like synoviocytes (FLSs), where LAMP*3* was shown to regulate epithelial–mesenchymal transition (EMT), cell proliferation, migration, and invasive behavior. Inhibition of *LAMP3* or EMT suppressed inflammatory and destructive phenotypes in RA-FLSs, and administration of an EMT inhibitor attenuated arthritis progression in a collagen-induced arthritis mouse model [16]. These findings suggest that LAMP3 may contribute to synovial activation and tissue remodeling under inflammatory conditions. Therefore, elevated *LAMP3* expression in female OA synovium may reflect enhanced synovial inflammatory remodeling and fibroblast activation, potentially contributing to sex-related differences in OA pathology.

MMD is a membrane-associated protein predominantly expressed in myeloid cells and implicated in macrophage differentiation and pro-inflammatory activation. In the present study, *MMD* expression remained significantly higher in the synovial tissue of female knee OA patients after adjustment for age, BMI, and KL grade, suggesting a potential role in sex-specific inflammatory regulation in knee OA. Inflammatory stimulation with LPS has been shown to upregulate *MMD* expression in macrophages, and *MMD* overexpression enhanced TNF-α and nitric oxide production through increased ERK1/2 and Akt phosphorylation, suggesting that MMD amplifies macrophage inflammatory activation [17]. In addition, PAQR11, also known as MMD, was strongly induced during monocyte-to-macrophage differentiation, and *Paqr11* knockdown or deletion attenuated macrophage differentiation. In vivo, *Paqr11* deficiency alleviated collagen-induced arthritis progression in mice, supporting its contribution to macrophage-related inflammatory arthritis [18]. Previous studies have demonstrated sex-dimorphic gene regulation and sex hormone-dependent immune regulation in macrophages and other innate immune cells, supporting the concept that macrophage-mediated inflammatory responses differ between females and males [19,20]. Therefore, the increased *MMD* expression observed in female knee OA synovium may reflect sex-dependent regulation of macrophage-mediated inflammation.

Sex-related differences in OA may also be influenced by changes in the hormonal milieu across the life course. Estrogens and other sex hormones can regulate immune and stromal cell functions, and menopausal hormonal changes have been associated with increased susceptibility to musculoskeletal pain and OA [20,21]. However, the effects of estrogen-related signaling on OA are likely to be tissue- and context-dependent, and the causal contribution of estrogen deficiency to OA pathogenesis remains unresolved [21,22]. In this context, the female-associated expression of *EGFL6*, *LAMP3*, and *MMD* may be influenced by direct and/or indirect effects of sex hormone-related signaling on angiogenic, inflammatory, and tissue-remodeling pathways. Notably, all female participants in the RNA-seq cohort (5/5, 100%) and 91% (71/78) of those in the qPCR cohort were aged ≥60 years, suggesting that most female participants were likely postmenopausal. However, because menopausal status, circulating hormone levels, and hormone replacement therapy were not assessed, the potential contribution of estrogen deficiency or other menopausal hormonal changes to the observed sex-related differences could not be evaluated directly. Prospective studies incorporating detailed reproductive history, hormonal measurements, and cell type-specific analyses are needed to determine how hormonal changes contribute to sex-related synovial alterations in OA.

Several additional female-upregulated genes identified by RNA-seq, including *COL6A6* and *CAPN6*, may also have biological relevance to OA pathology. *COL6A6* encodes a collagen VI chain involved in extracellular matrix organization and cartilage homeostasis. Previous genetic studies identified COL6A6 variants in patients with OA, suggesting a potential association between COL6A6 and OA susceptibility [23]. CAPN6 has also been implicated in inflammatory and tissue remodeling processes. Previous studies demonstrated that inflammatory cytokines induce CAPN6 expression in myoblasts and macrophages, and that CAPN6 contributes to persistent inflammatory lesions and inflammatory macrophage dysfunction under chronic inflammatory conditions [24,25]. Although *COL6A6* and *CAPN6* were no longer significant after adjustment, their elevated expression in females may reflect sex-related differences in extracellular matrix and inflammatory remodeling processes in OA synovium. While the precise roles of these genes in OA synovium remain unclear, their differential expression may provide additional insight into the molecular heterogeneity underlying sex differences in OA pathology.

In contrast, many male-upregulated genes identified by RNA-seq were Y chromosome-related transcripts, including *KDM5D*, *DDX3Y*, *ZFY*, *USP9Y*, *RPS4Y1*, *EIF1AY*, and *NLGN4Y*, reflecting chromosomal sex differences rather than OA-specific molecular mechanisms. After exclusion of these genes, *DAW1* emerged as a male-upregulated gene and became significantly associated with male sex after adjustment for confounding factors. DAW1 is known as a cilia-associated gene involved in axonemal dynein assembly and motile ciliary function [26]. Although its role in OA remains unclear, recent transcriptomic analyses in inflammatory disorders demonstrated reduced *DAW1* expression under inflammatory conditions [27], suggesting that inflammatory signaling may negatively regulate *DAW1* expression. In addition, primary cilia have increasingly been implicated in OA pathogenesis through regulation of mechanotransduction, inflammatory signaling, and cartilage homeostasis [28]. Therefore, higher *DAW1* expression in male OA synovium may reflect sex-related differences in cilia-associated homeostatic responses under inflammatory conditions.

Several genes, including CDK1 and FMO1, showed expression patterns that were inconsistent between the RNA-seq and qPCR analyses. The relatively small RNA-seq discovery cohort may have increased susceptibility to sampling variability and false-positive findings. In addition, RNA-seq and qPCR were performed in independent cohorts; therefore, differences in unmeasured clinical factors or inflammatory status may have contributed to between-cohort variability, despite the absence of major differences in age, BMI, or KL grade. Because synovial tissue is highly heterogeneous, variation in the relative abundance of synovial fibroblasts, infiltrating immune cells, and other cell populations may also influence bulk gene expression profiles. Furthermore, RNA-seq and qPCR quantify gene expression using different analytical approaches. RNA-seq captures transcript-level abundance across the transcriptome, whereas qPCR measures a defined amplicon; thus, transcript- or isoform-specific differences may contribute to platform-dependent results. Candidate selection from a small discovery dataset may also result in overestimation of effect sizes, followed by attenuation or non-replication in an independent validation cohort. Therefore, the discordant findings for CDK1 and FMO1 should be interpreted cautiously. Further validation in larger and clinically well-characterized cohorts, together with functional studies, is needed to clarify their potential sex-related roles in OA synovium.

Notably, age and KL grade were not significantly associated with expression of most candidate genes in the multivariable analyses, whereas BMI showed significant associations with *AZGP1* and *EGFL6* expression. These findings suggest that obesity-related metabolic or inflammatory factors may partially influence synovial gene expression independently of structural OA severity. Obesity is increasingly recognized not only as a mechanical risk factor but also as a systemic inflammatory condition contributing to OA pathophysiology through adipokine signaling and immune modulation.

This study has several limitations. First, the RNA-seq discovery cohort was relatively small, and all samples were obtained from a single institution, which may limit statistical power and generalizability. Second, both RNA-seq and qPCR were performed using bulk synovial tissue. Because OA synovium comprises heterogeneous cell populations, including synovial fibroblasts and infiltrating immune cells, the observed sex-related differences may reflect sex-dependent alterations in cellular composition in addition to cell-intrinsic transcriptional regulation. Bulk analysis may also mask cell type-specific changes in the direction or magnitude of gene expression and precludes precise assignment of the identified genes to specific cellular sources. Third, all samples were obtained from patients with established knee OA undergoing total knee arthroplasty, and healthy non-OA synovial tissue was not examined. Therefore, it remains unclear whether the identified signatures are specific to OA pathology, reflect baseline sex-related differences in synovial biology, or represent secondary alterations associated with advanced disease. Fourth, detailed information on menopausal status, hormone replacement therapy, medication use, comorbidities, and inflammatory status was not systematically available. Although the multivariable analyses were adjusted for age, BMI, and KL grade, residual confounding by these unmeasured factors cannot be excluded. Fifth, the present study focused exclusively on synovial tissue and did not assess subchondral bone, which may contribute to OA progression and pain. Thus, the relationship between sex-related synovial gene expression and subchondral bone pathology remains unknown. Finally, the findings were evaluated at the mRNA level only, and protein expression, cellular localization, and functional roles of the candidate genes were not examined. Future studies incorporating protein-level validation, single-cell or spatial transcriptomic analyses, and functional experiments in clinically well-characterized cohorts are needed to clarify the cellular sources and mechanistic significance of the identified sex-related synovial signatures.

## 5. Conclusions

In conclusion, the present study identified distinct sex-related synovial gene expression profiles in knee OA. In particular, *EGFL6*, *LAMP3*, and *MMD* were independently associated with female sex after adjustment for clinical confounders, suggesting potential sex-specific mechanisms in OA synovium. These findings provide new insight into the molecular basis of sex differences in OA and may contribute to the development of sex-specific therapeutic strategies for OA pathology.

## Figures and Tables

**Table 1:** Primer sequences used in this study.

Gene		Sequence	Base Pairs
*APCCD1L*	sense	GATGGAACAAGACCCCCTCG	97
	antisense	GGAGGTGGGACGTTTCTCTG	
*AZGP1*	sense	TGCAGGGAAGGTTTGGTTGT	145
	antisense	TTGGTTATCTGGGCTGCTGG	
*CAPN6*	sense	TTACCGCCGAATGGGAAGAC	108
	antisense	GCACGCTCCTGGATGTAGAG	
*CDK1*	sense	AACTACAGGTCAAGTGGTAGCC	74
	antisense	TTGCAGTACTAGGAACCCCTTC	
*COL6A6*	sense	TTGTGACCAAGCCAGACCAC	167
	antisense	CAGTGACAGAAGTCTCCGGC	
*C3orf80*	sense	AGAGCACGAGATGCGTGTAG	76
	antisense	GGTGGGCAGATACTTGACCT	
*DACT2*	sense	TCAGGACTATGGACGAGGCA	94
	antisense	AAAGGATGGGTGGGCAAAGT	
*DAW1*	sense	TGCTTGATCTTGGTCCCAGC	149
	antisense	CGTGTGATTGCTGTTCTGGC	
*DUOX2*	sense	ACCCCTGACTGTGCTTGAC	115
	antisense	ATAGGCCACCACTCCAGAGA	
*EGFL6*	sense	GCCAGATGCTACGTGTGTGA	87
	antisense	CACTGTGGCCCTTCTTCTGT	
*FMO1*	sense	CCTGCTTTGAGAGGAGCGAT	75
	antisense	GACTGGCTCTGCCTTCTTCA	
*LAMP3*	sense	TCAAACATGCGGTGGTGATG	200
	antisense	CCCAATCACAGGAAGCACAA	
*LEF1*	sense	ATCACACCCGTCACACATCC	132
	antisense	CCAAGAGGTGGGGTGATCTG	
*LRRN1*	sense	AAGTCCCTCAACTTGCCCTG	177
	antisense	TAGCGGTCGACAGAAACGAG	
*MMD*	sense	TGGACCCCTGGCATCTCATA	199
	antisense	AATTAAGCCCCCACAGGCAA	
*P2RY2*	sense	ACTGCTAAAGCCAGCCTACG	114
	antisense	TGGAATGGCAGGAAGCAGAG	
*USP2*	sense	CGAACCAGCAAGCTCACAAC	130
	antisense	TGGTGGTTCCGGAGTGATTG	

**Table 2:** Patient demographic data.

	RNA-Seq			qPCR		
	Female (*n* = 5)	Male (*n* = 5)	*p*-Value	Female (*n* = 78)	Male (*n* = 27)	*p*-Value
Age (y)	77 (67–85)	72 (54–85)	0.469	73.5 (44–92)	76 (51–91)	0.381
BMI (kg/m^2^)	22.2 (19.6–27.4)	23.7 (18.8–30.8)	0.677	27.7 (17.9–45.3)	25.8 (17.4–56.7)	0.194
KL grade 2	0 (0.0)	0 (0.0)	1.000	3 (3.8)	3 (11.1)	0.228
KL grade 3	2 (40.0)	1 (20.0)		20 (25.6)	9 (33.3)	
KL grade 4	3 (60.0)	4 (80.0)		55 (70.5)	15 (55.6)	

Continuous variables are presented as median (range), and categorical variables are presented as n (%). Normality was assessed using the Shapiro–Wilk test. *p*-values for age were calculated using Student’s *t*-test. *p*-values for BMI were calculated using Student’s *t*-test in the RNA-seq cohort and the Mann–Whitney U test in the qPCR cohort. KL grade distributions were compared using Fisher’s exact test. BMI, body mass index; KL, Kellgren–Lawrence.

**Table 3:** Differentially expressed genes in females and males identified by RNA-seq.

Upregulated Genes in Females			Upregulated Genes in Males		
Genes	log2FC	q-Value	Genes	log2FC	q-Value
*APCDD1L*	2.19	1.61 × 10^−2^	*DACT2*	2.00	3.30 × 10^−4^
*AZGP1*	2.22	4.59 × 10^−2^	*DAW1*	2.46	1.44 × 10^−5^
*C3orf80*	1.38	4.23 × 10^−3^	*DDX3Y*	5.11	1.28 × 10^−4^
*CAPN6*	2.00	1.19 × 10^−2^	*DUOX2*	2.34	3.49 × 10^−6^
*CDK1*	1.02	1.24 × 10^−2^	*EIF1AY*	5.34	9.64 × 10^−15^
*COL6A6*	2.11	4.75 × 10^−2^	*KDM5D*	5.28	2.04 × 10^−6^
*EGFL6*	2.69	1.38 × 10^−2^	*NLGN4Y*	5.73	1.04 × 10^−17^
*FMO1*	1.38	2.48 × 10^−2^	*ENSG00000266302*	1.20	2.33 × 10^−5^
*LAMP3*	1.98	3.03 × 10^−2^	*P2RY2*	1.13	7.06 × 10^−4^
*LEF1*	1.27	4.22 × 10^−3^	*RPS4Y1*	5.22	4.23 × 10^−3^
*LRRN1*	2.02	2.63 × 10^−2^	*USP2*	1.13	3.67 × 10^−4^
*MMD*	1.09	1.24 × 10^−2^	*USP9Y*	5.40	1.45 × 10^−5^
			*ZFY*	5.03	2.38 × 10^−15^

**Table 4:** Multivariable linear regression analysis of gene expression adjusted for BMI, age, and Kellgren–Lawrence grade.

Variable	SEX		BMI		Age		KL	
	*p*-Value	β	*p*-Value	β	*p*-Value	β	*p*-Value	β
APCDD1L	0.374	0.143	0.142	0.311	0.465	0.112	0.853	0.054
AZGP1	0.073	0.435	0.021	0.639	0.598	0.084	0.722	0.064
CAPN6	0.088	0.401	0.052	0.494	0.993	0.05	0.268	0.197
CDK1	0.021	0.644	0.537	0.094	0.414	0.128	0.053	0.492
COL6A6	0.387	0.138	0.228	0.225	0.785	0.058	0.719	0.065
C3orf80	1.000	0.050	0.433	0.122	0.951	0.050	0.231	0.222
DACT2	0.205	0.244	0.859	0.054	0.654	0.073	0.582	0.085
DAW1	0.009	0.753	0.494	0.104	0.720	0.065	0.14	0.313
DUOX2	0.951	0.500	0.631	0.076	0.418	0.127	0.718	0.065
EGFL6	0.004	0.829	0.049	0.506	0.336	0.160	0.123	0.338
FMO1	0.005	0.812	0.729	0.064	0.361	0.149	0.644	0.074
LAMP3	0.029	0.596	0.28	0.189	0.753	0.061	0.244	0.213
LEF1	0.103	0.370	0.466	0.112	0.231	0.076	0.739	0.063
LRRN1	0.868	0.053	0.312	0.171	0.755	0.061	0.471	0.111
MMD	0.014	0.698	0.236	0.219	0.105	0.368	0.940	0.051
P2RY2	0.141	0.313	0.238	0.217	0.983	0.050	0.890	0.052
USP2	0.259	0.202	0.375	0.143	0.643	0.075	0.085	0.406

BMI; body mass index, KL, Kellgren–Lawrence grade.

## Data Availability

The data presented in this study are available on request from the corresponding author.

## Abbreviations

The following abbreviations are used in this manuscript:

OA	Osteoarthritis
RNA-seq	RNA sequencing
DEGs	Differentially expressed genes
qPCR	quantitative PCR
BMI	Body mass index
KL	Kellgren–Lawrence
CGRP	Calcitonin gene-related peptide
RA	Rheumatoid arthritis
FLSs	Fibroblast-like synoviocytes
EMT	Epithelial–mesenchymal transition
